# Long-circulating XTEN864-annexin A5 fusion protein for phosphatidylserine-related therapeutic applications

**DOI:** 10.1007/s10495-021-01686-w

**Published:** 2021-08-17

**Authors:** Akvile Haeckel, Lena Ascher, Nicola Beindorff, Sonal Prasad, Karolina Garczyńska, Jing Guo, Eyk Schellenberger

**Affiliations:** 1 Department of Radiology, Charité – Universitätsmedizin Berlin, Charitéplatz 1, 10117 Berlin, Germany; 2 Berlin Experimental Radionuclide Imaging Center (BERIC), Charité – Universitätsmedizin Berlin, Augustenburger Platz 1, 13353 Berlin, Germany; 3 Department of Nuclear Medicine, Charité – Universitätsmedizin Berlin, Augustenburger Platz 1, 13353 Berlin, Germany; 4 Bundesanstalt für Materialforschung und -prüfung (BAM), Richard-Willstätter-Straße 11, 12489 Berlin, Germany

**Keywords:** Programmed cell death, Apoptotic mimicry, Atherosclerosis, Inflammation, Inflammatory cytokine storm, Cancer

## Abstract

Annexin A5 (anxA5) is a marker for apoptosis, but has also therapeutic potential in cardiovascular diseases, cancer, and, due to apoptotic mimicry, against dangerous viruses, which is limited by the short blood circulation. An 864-amino-acid XTEN polypeptide was fused to anxA5. XTEN864-anxA5 was expressed in *Escherichia coli* and purified using XTEN as tag. XTEN864-anxA5 was coupled with DTPA and indium-111. After intravenous or subcutaneous injection of ^111^In-XTEN864-anxA5, mouse blood samples were collected for blood half-life determination and organ samples for biodistribution using a gamma counter. XTEN864-anxA5 was labeled with 6S-IDCC to confirm binding to apoptotic cells using flow cytometry. To demonstrate targeting of atherosclerotic plaques, XTEN864-anxA5 was labeled with MeCAT(Ho) and administered intravenously to atherosclerotic ApoE^−/−^ mice. MeCAT(Ho)-XTEN864-anxA5 was detected together with MeCAT(Tm)-MAC-2 macrophage antibodies by imaging mass cytometry (CyTOF) of aortic root sections. The ability of anxA5 to bind apoptotic cells was not affected by XTEN864. The blood half-life of XTEN864-anxA5 was 13 h in mice after IV injection, markedly longer than the 7-min half-life of anxA5. 96 h after injection, highest amounts of XTEN864-anxA5 were found in liver, spleen, and kidney. XTEN864-anxA5 was found to target the adventitia adjacent to atherosclerotic plaques. XTEN864-anxA5 is a long-circulating fusion protein that can be efficiently produced in *E. coli* and potentially circulates in humans for several days, making it a promising therapeutic drug.

**Supplementary Information:**

The online version contains supplementary material available at 10.1007/s10495-021-01686-w.

## Introduction

Phosphatidylserine (PS) is the most abundant negatively charged phospholipid in eukaryotic membranes. PS directs the binding of proteins that bear C2 or gamma-carboxyglutamic domains and contributes to the electrostatic association of polycationic ligands with cellular membranes. PS preferably occurs in the inner leaflet of the plasma membrane and endocytic membranes. Loss of PS asymmetry is a well-regulated process and an early indicator of programmed cell death (apoptosis) and is a signal to initiate blood clotting [1, 2]. In hemostasis, platelets exposing PS regulate the coagulation process and coordinate the attachment and activation of some clotting factors and activate thrombin production [3]. Furthermore, externalized PSs on cells act as key modulators for the highly conserved immunosuppression during normal homeostasis: they do not only serve as early signals for apoptosis, but their exposure is also recognized by phagocytes, such as macrophages, dendritic cells, or epithelial cells, which clear apoptotic cells via engulfment (efferocytosis) before they lose their membrane integrity and release enzymes, oxidants, and other irritative and toxic components into surrounding tissue, thereby preventing any significant inflammation [4–7]. However, dysregulation of PS externalization can cause or is involved in numerous acute and chronic diseases and syndromes that can cause inflammation, thrombosis, and bleeding (myocardial ischemia, systemic hemorrhage, sepsis, atherosclerosis), facilitating the spread of bacterial and viral infection or inhibiting, or rather influencing, the immune response (cancer, autoimmune diseases) [1, 4, 6, 8, 9].

Annexin A5 (anxA5) belongs to the large family of mostly intracellularly expressed annexin proteins and reversibly binds Ca^+2^-dependently with high affinity to PS-exposing cell membranes [10]. AnxA5 has been widely used for detecting apoptotic processes in vitro and in vivo using different imaging techniques, such as radionuclide-based imaging [11], optical imaging [12–14], magnetic resonance imaging [15–17], and ultrasound [18, 19].

Besides its role in detecting apoptosis, anxA5 appears to have promising properties for various therapeutic purposes, as suggested by recent studies. Many enveloped viruses (e.g., vaccinia virus, dengue virus, Ebola virus, and pseudotyped lentivirus) rely on what is known as apoptotic mimicry to promote their infectious entry and replication in host cells as well as for immune evasion [20]. Such viruses use different strategies to acquire host cell phosphatidylserine and incorporate it into the viral membrane. These apoptotic body-like, PS-exposing viruses in turn can be recognized and internalized by host cells with PS receptors [20, 21]. Consequently, masking vaccinia viruses with anxA5 was shown to reduce infectivity by 90% [22]. Moreover, for Ebola, anxA5 was hypothesized to cloak the PS-exposing envelop, thus preventing the pathologic inflammatory cytokine storm and hemorrhagic consumptive coagulopathy [23], which might also be the case for other viruses including SARS-CoV-2 [24, 25].

Besides its diagnostic and therapeutic potential in the important field of viral infection, anxA5 has shown promising therapeutic effects in several other areas as well. For cardiovascular disease, anxA5 was reported to reduce inflammation in advanced atherosclerotic plaques and to attenuate plaque progression in early disease [26, 27]. Furthermore, anxA5 reduced vessel inflammation and atherosclerosis after arterial cuff placement or vein graft surgery [28]. In myocardial ischemia–reperfusion injury, anxA5 was shown to suppress the inflammatory response and reduce infarction size [29]. A dimeric anxA5, administered after the onset of hemorrhagic shock or sepsis, was able to protect the lungs, kidneys, and gut from massive dysfunction [30, 31].

Recently, it has been demonstrated in mice that anxA5 countered immunosuppression induced by chemotherapy in peritumoral tissues, enhanced general anti-tumor efficacy and thus could, in combination with cisplatin and a human papillomavirus 16 (HPV-16)-derived E7 peptide, reverse HPV-16-positive cervical tumor growth and let the mice survive [32].

Regarding the pathogenesis of Alzheimer’s disease, Bartolome et al. reported that anxA5 protected choroid plexus cells from amyloid (Aβ)-induced toxicity [33]. These cells are part of a special brain structure that produces cerebrospinal fluid (CSF). Interestingly, interaction with anxA5 also reduced misfolding and fibril formation of alpha-synuclein, which plays a key role in the pathogenesis of Parkinson’s disease [34].

An important obstacle for the clinical use of anxA5 as a therapeutic drug is its very short blood half-life of 24 min after injection in humans, since it is freely filtrated, excreted and accumulated by the kidneys [35]. The relatively costly production of wild-type anxA5 by expression and purification in *Escherichia coli* precludes its frequent or continuous administration. Therefore, a long-circulating and effectively producible anxA5 is highly desirable.

Previously, we modified anxA5 by fusion with the XTEN288 polypeptide and were thus able to prolong blood circulation time in mice from 7 min to 1 h, which in turn allowed improved imaging of apoptosis in cancer treatment [36]. XTEN is an unstructured polypeptide that binds a large water shell, which prolongs blood circulation after injection and stabilizes proteins that are fused to XTEN. It is composed of a randomized sequence of six different amino acids comprising about 8% alanine, 12% glutamic acid, 18% glycine, 17% proline, 28% serine, and 17% threonine [37]. XTEN provides similar properties and several crucial advantages in comparison to polyethylene glycol (PEG), a well-known synthetic polymer used to prolong the blood circulation of both therapeutic and diagnostic molecules: fused with the protein on the DNA level, XTEN results in a fusion protein that can be expressed recombinantly, makes the fused protein heat-stable and soluble, and serves as a purification tag itself, thus avoiding the need to cleave off e.g. His tags. Unlike PEG, which should only be used at sizes below 5 kDa to ensure adequate excretion, XTEN is biodegradable and nonimmunogenic and thus can be used in longer peptide lengths [37–39]. Although PEG has low immunogenicity and is considered nontoxic, some animal studies have demonstrated renal vacuolation following administration of PEGylated proteins in mice [40] and antibody production against PEG in mice [41, 42] and dogs [43]. PEG is assumed to induce antibody production not only after therapeutic administration but also after prolonged use of PEG-containing cosmetics [42]. Several clinical trials in different phases are currently investigating XTEN proteins fused to growth hormones or clotting factors for the treatment of growth disorders and hemophilia.

Here, we describe the design and characterization of the new recombinant XTEN864-anxA5 synthesized by fusing with a long XTEN consisting of 864 amino acids. This new molecule was synthesized to substantially extend the blood half-life for therapeutic applications and to add other beneficial properties of XTEN [37].

## Materials and methods

Unless specified otherwise, reagents were purchased from Merck KGaA (Darmstadt, Germany).

### Expression and purification of XTEN864-anxA5

We designed, expressed, and purified the XTEN864-anxA5 fusion protein largely following the procedure described before [44], with some modifications in the protocol. Briefly, an XTEN sequence of 864 amino acids (XTEN864)[37] was joined to complete DNA of the human anxA5 gene (NP 1145.1). One cysteine was added at the first N-terminal position to allow subsequent coupling. The endogenous cysteine of anxA5 at position 316 was changed to serine to avoid unspecific labeling (for sequence see Supplementary information). Two stop codons were added to eliminate C-terminal tag expression of the pET30a(+) plasmid. Gene synthesis was performed by Genscript USA Inc. The protein sequence of XTEN864-anxA5 is given in the Supplementary section.

Heat shock–competent *E. coli* BL21(DE3) Gold Cells (Agilent Technologies) were transformed with the pET30(+) plasmid–carrying the sequence of the fusion protein and spread to grow on agar plates containing kanamycin (100 μg/ml). A single colony was picked to produce 10 ml of culture overnight in LB (Luria–Bertani) medium with 100 µg/ml kanamycin growing at 32 °C. Further, the overnight culture was diluted 1:40 to inoculate 0.4 l of MagicMedia (Life Technologies) *E. coli* expression medium for the main culture. This culture was allowed to grow at 32 °C with 300 rpm for 7 h and was further cultivated at 32 °C for 24 h with 275 rpm shaking intensity.

Bacterial cells were then collected by centrifugation, and 7.5 g of wet cell pellet were lysed in 35 ml of PER-B protein extraction reagent (Thermo Fisher) containing DNAse I and lysozyme at the recommended concentrations and Proteinase Halt protease inhibitor cocktail (Thermo Fisher). The bacterial cell lysate was loaded onto a 50-ml weak anion exchange column with diethylaminoethyl (DEAE) cellulose, equilibrated with starting buffer (20 mM Tris, 50 mM NaCl, pH 6.8). The protein of interest was eluted with a gradient to end buffer (20 mM Tris, 0.5 M NaCl, pH 6.8) with a flow rate of 3 ml/min using a BioLogic LP system (BioRad). The fractions containing the fusion protein were determined by sodium dodecyl sulfate polyacrylamide gel electrophoresis (SDS-PAGE) (Novex 4%–12% Bis–Tris gradient gel; Life Technologies) with subsequent Coomassie Simply Blue SafeStain (Life Technologies) and then pooled, and the elution buffer was exchanged against equilibration buffer (20 mM Tris, 50 mM NaCl, pH 6.8). The solution was loaded onto a 50-ml strong anion exchange column with UnosphereQ (BioRad) (equilibrated with 20 mM Tris, 50 mM NaCl, pH 6.8), and the protein of interest was again eluted using a gradient to end buffer (20 mM Tris, 1 M NaCl, pH 6.8) with a flow rate of 3 ml/min. XTEN864-anxA5 fractions were selected as described above and then pooled, and NaCl concentration of the buffer was increased to 2.5 M. This solution was added to a 30-ml Octyl-Sepharose 4 Fast Flow (Sigma-Aldrich) hydrophobic interaction column, which was equilibrated with high salt buffer (20 mM Tris, 3 M NaCl, pH 7.5), and the desired protein was eluted using a decreasing gradient to end buffer (20 mM Tris, 135 mM NaCl, pH 7.5). XTEN864-anxA5 fractions were selected again as described above, then pooled, and desalted against storage buffer (10 mM HEPES [(4-(2-hydroxyethyl)-1-piperazineethanesulfonic acid)], 135 mM NaCl, pH 7.5). The protein solution was passed through a 0.22-μm filter, and the concentration of XTEN864-anxA5 was measured using a BCA protein assay applying a correction factor of 1.8 (adapted from Haeckel et al. 2014) [36].

### Coupling of XTEN864-anxA5 with 6S-IDCC- and DTPA-maleimides and labeling of XTEN864-anxA5-DTPA with ^111^In^3+^

Thiol-directed coupling of XTEN864-anxA5 with diethylenetriamine pentaacetic acid (DTPA) (CheMatech, France) and 6S-IDCC (Mivenion, Germany) maleimides was performed as described before [36].

An aliquot of XTEN864-anxA5-DTPA (300 µg) was mixed 1:1 (v/v) with reaction buffer (270 mM sodium acetate, 80 mM Gentisic acid, pH 5.0), and the resulting solution was then added to ^111^InCl_3_ solution in 0.02 M HCl (233 MBq) and incubated for 70 min at room temperature. Thereafter, the radiolabeled protein was purified by 2–3 rounds of ultrafiltration with 10 kDa Amicon Ultra 0.5 centrifugal filters (Millipore) by adding 0.45 ml of washing buffer (10 mM HEPES, 140 mM NaCl, pH 7.4) after every round. Radioactivity in the filtration unit and filtrate was measured after each ultrafiltration step. The labeling procedure yielded 71 MBq of ^111^In^3+^-XTEN864-anxA5 ready for injection in mice. Radiochemical purity determined using TLC was ≥ 90%. The radiochemical yield was 30%. All solutions were freshly prepared directly before each radiolabeling session.

### Labeling of XTEN864-anxA5 and Mac-2 antibody with MeCAT

Labeling was done using the metal-coded affinity tag (MeCAT, Proteome Factory AG) technique, which is based on chelates loaded with different lanthanides. 100 µg protein were used for the labeling. First, the protein solution was transferred to a HEPES buffer. For this, the protein solution to be labeled was centrifuged three times with 400 µl buffer for 5 min at 14,000 g in 10 kDa centrifuge filters (Eppendorf AG) at 4 °C. Centrifugation was followed by partial reduction of disulfide bridges. For this, the buffered protein solution was incubated with 100 μl of a freshly prepared 4 mM TCEP solution for 30 min at 37 °C on a shaker (Thermomixer Comfort, Eppendorf AG). The reducing agent was then washed out by centrifuging the solution twice with 400 μl buffer for 5 min at 14,000 g in centrifuge filters at 4 °C. The reduced protein was adjusted to 100 µl with the buffer used. The MeCAT (XTEN864-anxA5: Holmium; Mac-2 (Cedarlane, Canada): Thulium) is thawed and dissolved in 25 µL 5 mM sodium acetate, pH 5.3, and 5 µl of this solution was incubated with the protein solution for 60 min at 37 °C on a shaker (Thermomixer Comfort, Eppendorf). The excess MeCAT was then washed out by centrifuging the solution twice with 400 µl buffer for 5 min at 14,000 g in centrifuge filters at 4 °C. For quantification of the yield, the volume of the labeled protein solution and the concentration were measured spectrometrically at 280 nm (Nano Drop 2000 UV–Vis spectrophotometer, Thermo Scientific).

### Western blot analysis

Proteins were loaded onto 4–12% Bis–Tris Novex gradient gel, and SDS-PAGE was performed under recommended conditions. After electrophoresis, the proteins were transferred onto nitrocellulose membrane using iBlot gel transfer system (Life Technologies, Darmstadt, Germany). The membrane was stained with rabbit annexin V antibody (1:1000, ab14196, Abcam, Cambridge, UK) and developed using the WesternBreeze® chromogenic Western blot immunodetection kit.

### Flow cytometry

Human T-cell leukemia (Jurkat) cells were purchased from the Leibniz Institute DSMZ-German Collection of Microorganisms and Cell Cultures and grown in RPMI 1640 medium containing GlutaMAX, supplemented with 10% fetal bovine serum, 100 U/ml penicillin, and 100 mg/ml streptomycin, at 37 °C with 5% CO_2_. To induce apoptosis, the cells were treated with camptothecin at a final concentration of 9 mM for 5 h at 37 °C with 5% CO_2_, then harvested, centrifuged at 194 × g for 5 min, and washed in cold phosphate-buffered saline (PBS, pH 7.4) with 1% fetal bovine serum. After a repeated centrifugation step, the cells were resuspended in cold binding buffer (1.8 mM CaCl_2_, 10 mM HEPES, 150 mM NaCl, 5 mM KCl and 1 mM MgCl_2_, pH 7.4) and aliquoted at a density of 5 × 10^5^ / 500 μl. Further, the cells were incubated with 0.2 μg XTEN-anxA5-6S-IDCC or wild-type anxA5-FITC (FITC Annexin V Apoptosis Detection Kit I, BD Biosciences) for 30 min in the dark on ice. Subsequently, cells were centrifuged again, the supernatant was removed, and cells were fixed for 30 min with 1% paraformaldehyde at room temperature. Afterwards, paraformaldehyde was exchanged against binding buffer. Measurements were performed using a BD Accuri® C6 flow cytometer (BD Biosciences) at an excitation length of λ = 488 nm (FITC) and λ = 633 nm (NIRF dye). Results were analyzed using BD Accuri® C6 Analysis software (BD Biosciences).

### SPECT/CT imaging of ^111^In-XTEN864-anxA5 and determination of blood half-life

All animal procedures were performed according to Berlin State Office for Health and Social Affairs-approved animal welfare guidelines (approval number G0176/17). Eight-week-old Balb/c female mice were obtained from Charles River Laboratories (Sulzfeld, Germany) and fed with normal chow diet for at least one week prior to experiments. Anesthesia was induced and maintained with 1–1.5% isoflurane throughout the imaging procedure. The mice were injected with approximately 100 MBq of 111In-XTEN864-anxA5 (80 μl) via the tail vein or subcutaneously. The body temperature was maintained throughout the experiments with a warming bed. Single-photon emission computed tomography (SPECT) imaging combined with computed tomography (CT) was performed at four time points in a NanoSPECT/CTplus scanner (Mediso, Hungary/Bioscan, France).

In vivo blood half-life determination was done using an automatic gamma counter (1480 Wizard 3, Wallac). For this, samples of approx. 3 µl blood were taken from each mouse at different time points using a thin tail vein catheter (0.28 × 61 mm) with a 30G needle. The whole catheter was placed in the counter tube. The blood half-life after IV injection of 6 mice was calculated by fitting the means of the percentages of the injected activity per gram [%IA/g] with a one-phase exponential decay model using Prism 8 software (GraphPad Software Inc.). The subcutaneous injections of 5 mice were analyzed accordingly employing a two-compartment model {Y = A*[exp(− K2*t) − exp(− K1*t)]}. For further ex vivo investigation of biodistribution, the animals were sacrificed under anesthesia after all blood samples were taken. The retrieved organs were weighed and measured in a gamma counter device.

### In vivo administration of XTEN864-anxA5 to an atherosclerosis mouse model and tissue preparation for Hyperion imaging mass spectrometry

All animal procedures were performed according to Berlin State Office for Health and Social Affairs-approved animal welfare guidelines (approval number G0176/17). The ApoE knock-out mice were fed with western-type diet (Altromin, Germany) for 12 weeks. 10 mg/kg body weight ^111^In-XTEN864-anxA5 was administered intravenously into the tail vein, and the mice were sacrificed 24 h after injection by an overdose of isoflurane in combination with xylazine. The organs were perfused with HEPES buffer containing a high calcium concentration to avoid dissociation of XTEN864-anxA5 from binding sites (10 mM HEPES, 140 mM NaCl, 2.5 mM CaCl_2_, pH = 7.4). The aorta, heart, and other organs were fixed in the same buffer supplemented with 4% paraformaldehyde.

### Tissue staining

Aortic root samples were cut into 5 µm tissue sections and, mounted on glass slides for conventional staining procedures and imaging mass spectrometry.

First, the tissue sections were dewaxed with xylene and rehydrated in a descending alcohol series from 100% ethanol to 70% ethanol and finally Millipore water. Then the tissue sections were heated in a microwave oven in 10 mM citrate buffer, pH 6.0, for 20 min, while maintaining the temperature below the boiling point. After cooling for 15 min, the tissue sections were washed in PBS buffer, blocked for 10 min using Protein Block (Abcam), and then washed again with PBS.

For detection of XTEN864-anxA5 and macrophages on the same section, tissue was incubated with MeCAT(Tm)-labeled Mac-2 antibody (Cedarlane, Canada, labeling procedure described above) diluted in block solution at an optimized concentration (0.05432 µg/µl) for 1 h at RT. After repeated washing with PBS, nuclei were stained with the diluted DNA intercalator ^193^Iridium (1:400, Fluidigm) for 30 min. Finally, the slides were washed with water (Millipore) for 5 min and air-dried. For staining of parallel sections with unlabeled Mac-2 (Cedarlane, Canada, 1:1000), standard protocols using biotinylated secondary antibody (rabbit anti-rat ab6734, 1:1000, Abcam) and the DAB Kit (Abcam) were applied. H&E stain was performed using ready-to-use solutions from Roth, Germany (Mayer’s Hematoxylin, T865.1 and G-Eosin 0,5%, X883.2). All images were acquired with a fluorescence microscope (Zeiss Observer.Z1, Carl Zeiss AG).

### Imaging mass spectrometry instrumentation and measurement

Measurements were performed using the Hyperion imaging system (Fluidigm). This technique enables the identification of element distributions in prepared histological sections. Many elements can be spatially resolved in one section during a single measurement.

A laser power test was carried out before each measurement. In addition, tuning on a 3-Element Full Coverage Tuning Slide (PN 201,088) ^175^Lu was performed before each measurement to optimize helium flow. Measurements were carried out at 200 Hz frequency. The MCD Viewer (Fluidigm) was used for evaluation. Images were postprocessed with ImageJ (National Institutes of Health NIH, USA). For noise reduction, the build-in Gaussian Blur filter (Sigma = 2) of ImageJ was applied for red and green channel.

## Results

### pET-XTEN864-anxA5 plasmid achieves good expression in *E. coli* and can be purified using XTEN as affinity tag

**Fig. 1:**
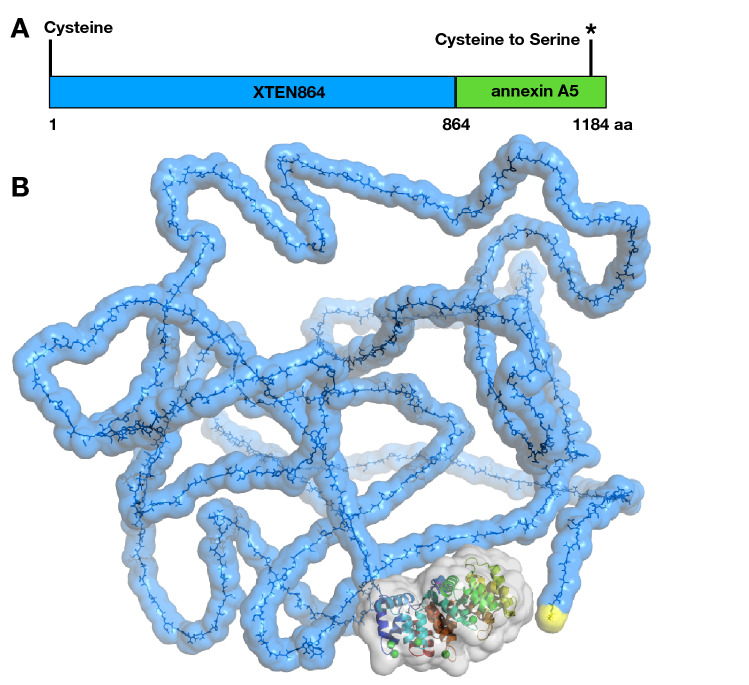
Schematic construct **A** and molecular model **B** of the XTEN864-anxA5 fusion protein. The DNA/protein sequence of the fusion protein starts with an N-terminal cysteine (yellow) for specific thiol-directed labeling reactions, followed by the long unstructured polypeptide XTEN864 (blue) fused to anxA5 protein (colored) at the C-terminus. AnxA5 includes chelated calcium atoms (green spheres) that are essential for binding to phosphatidylserine-exposing cell membranes, e.g., of apoptotic cells. An endogenous cysteine was point-mutated to serine to prevent unwanted thiol reactions with the anxA5 part (Color figure online)

Transfection of *E. coli* with pET-XTEN864-anxA5 plasmid resulted in a good yield and purity of expressed XTEN864-anxA5. The fusion DNA/protein is characterized by an N-terminal cysteine for specific, thiol-directed labeling reactions, a long XTEN consisting of 864 amino acids, and a human anxA5 with a point mutation of cysteine at position 316 to serine to prevent thiol-coupling within the anxA5 protein (Fig. 1). When lysozyme was added to the lysis solution, we obtained a lysate with a higher amount of host cell proteins but also with a markedly larger amount of XTEN864-anxA5 in the soluble fraction.

**Fig. 2:**
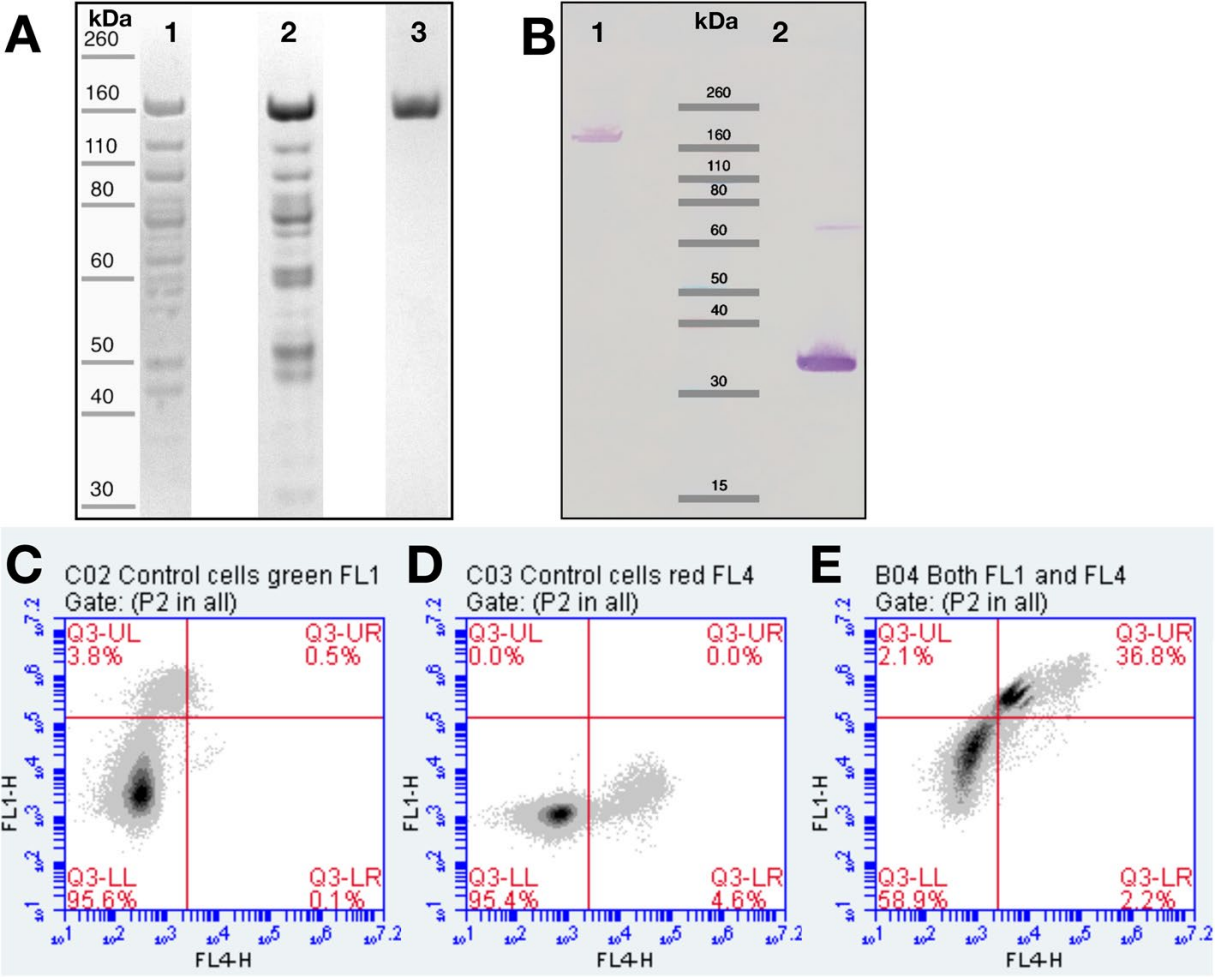
Purification with affinity columns, proof of identity and functionality of XTEN864-anxA5 in vitro. After expression and cell disruption, XTEN864-anxA5 was purified using XTEN as purification tag. **A** SDS-PAGE gel electrophoresis of the XTEN864-anxA5 fusion protein (apparent size, 160 kDa) after the first purification step using a weak anion exchange column (DEAE, lane 1), second step with a strong anion exchange column (UnoQ, lane 2), and after the third step using a hydrophobic interaction chromatography column (OctylS, lane 3). **B** Western blot analysis of purified XTEN864-anxA5 (3.4 µg, lane 1) and human cys-anxA5 (1 µg, lane 2) stained with anti-anxA5 antibodies. Note that, due to size and other properties of XTEN, XTEN864-anxA5 (115.3 kDa) stains substantially weaker in comparison to anxA5 (35.7 kDa). Partially formed anxA5-dimers through oxidation of N-terminal cysteine are visible at 71 kDa. **C**–**E** Comparison of binding properties of XTEN864-anxA5 with human anxA5 to apoptotic cells determined by flow cytometry. Panel **C**, **D** represents untreated Jurkat control cell population with typical low apoptosis level stained either with fluorescein-anxA5 (FL1) (**C**) or with 6S-IDCC-XTEN864-anxA5 (FL4) (**D**). **E** Camptothecin-treated Jurkat T cells were incubated with a mixture of equal-molar fluorescein-anxA5 and 6S-IDCC-XTEN864-anxA5 fusion protein. Cells were either negative (lower left quadrant, 58.9%) or positive (upper right quadrant, 36.8%) for both, confirming comparable binding capability of anxA5 and XTEN864-anxA5 for apoptotic cells

The target fusion protein was purified (Fig. 2A) from bacterial cell lysate utilizing the glutamic acid-rich XTEN864 as affinity tag with a weak (DEAE) followed by a strong (UnoQ) anionic exchange column. In the third step, binding of the anxA5 part to a hydrophobic interaction column (Octyl Sepharose) was used to remove possible XTEN fragments as described above. A rebuffered, concentrated and filter-sterilized solution of purified XTEN-anxA5 (Fig. 2B) was further used for different labeling procedures at the N-terminal cysteine residue.

The final protein yield from 0.4 l bacterial culture in autoinduction media (see also Materials and Methods) was approx. 10 mg as measured by BCA® assay using a correction factor of 1.8, which is required for XTEN fusion proteins due to the absence of aromatic residues in the XTEN sequence [36]. Purified XTEN864-anxA5 was further characterized by Western blotting (Fig. 2B), showing good binding capability of antibodies against wild-type anxA5 and the fusion protein. The staining intensity of XTEN864-anxA5 was lower than that of wild-type anxA5, as described for XTEN288-anxA5[36], which is probably attributable to interference of XTEN with the antibody and impaired transfer and binding to the membrane during the blotting procedure [37].

### Site-specific labeling of XTEN864-anxA5 on N-terminal cysteine residue

XTEN864-anxA5 was coupled with maleimide-DTPA for radionuclide labeling (^111^Indium), with the near-infrared fluorescence (NIRF) dye maleimide-6S-IDCC for optical detection, or with Holmium metal-containing MeCAT(Ho)-maleimide for imaging with a Hyperion mass spectrometer.

The labeling efficiency of XTEN864-anxA5 (protein concentration measured by BCA® Assay) with maleimide-6S-IDCC was 78% (extinction coefficient of 240,000 l mol^−1^ cm^−1^ at 682 nm) and of DTPA-XTEN864-anxA5 with the radionuclide ^111^Indium 30%.

### XTEN864-anxA5 binds phosphatidylserine-expressing apoptotic cell membranes

Cultured Jurkat T-cells were treated with camptothecin to induce apoptosis for comparison of the binding affinity of anxA5 and XTEN864-anxA5. Untreated control cells that were incubated with fluorescein isothiocyanate (FITC)-labeled wild-type anxA5 (green, Fig. 2C) and with 6S-IDCC-labeled XTEN864-anxA5 (NIRF, Fig. 2D), had a basal rate of 2–5% apoptotic/necrotic cells in flow cytometry. Investigation of camptothecin-treated cells incubated with an equal molar mixture of FITC-anxA5 and 6S-IDCC-XTEN864-anxA5 showed that both anxA5 variants bind to the same PS-exposing apoptotic Jurkat T cells (37%) with good affinity (Fig. 2E).

### SPECT/CT imaging of XTEN864-anxA5 and determination of blood circulation time

**Fig. 3:**
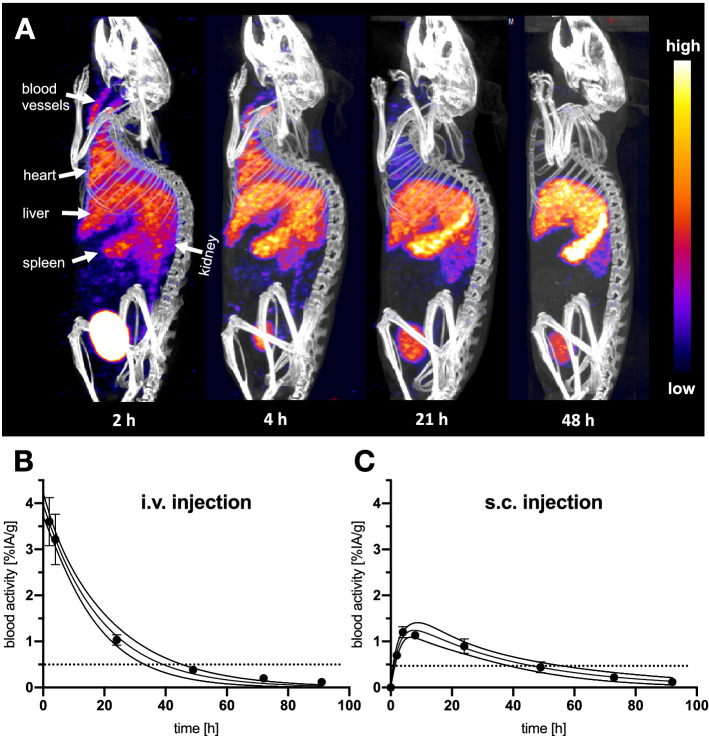
SPECT/CT imaging of XTEN864-anxA5 over time and determination of blood half-life in mice by in vivo measurement. **A** SPECT/CT of ^111^In-XTEN864-anxA5 at four time points visualizes long blood circulation and accumulation/excretion by liver, spleen, and kidneys after IV administration. **B** Time course of blood circulation of ^111^In-XTEN864-anxA5 after IV (6 mice) and **C** subcutaneous injection (5 mice) was determined by drawing tiny amounts of blood and measurement in a gamma counter (mean and SEM of percentage of the injected activity per gram blood [%IA/g], fitted curves with 95% confidence bands)

**Fig. 4:**
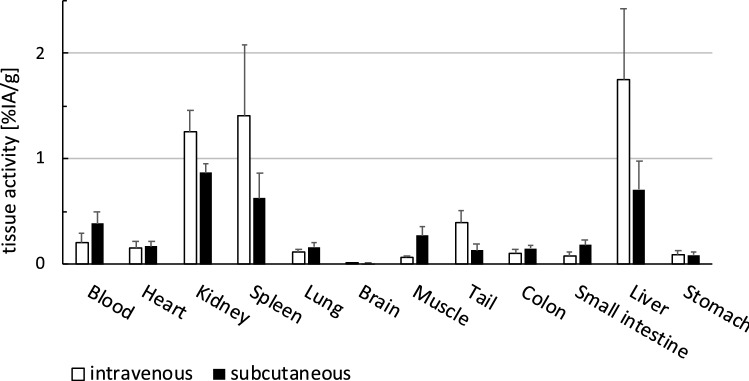
Biodistribution of XTEN864-anxA5 in mice four days after injection. Distribution of ^111^In-XTEN864-anxA5 in organ tissues (percentage of injected activity per gram blood [%IA/g]) was determined ex vivo by gamma counter measurement 4 days after IV or subcutaneous injection. XTEN864-anxA5 was mainly detectable in liver, spleen, kidney, and blood. Blood and muscle levels in mice after four days were still higher after subcutaneous injection than after IV injection

^111^In-DTPA-XTEN864-anxA5 was injected into the tail vein or under the skin of wild-type mice (Fig. 3). Figure 3A shows SPECT/CT images of the body distribution of ^111^In-DTPA-XTEN864-anxA5 in vivo over 48 h. The fusion protein was cleared from blood mainly by the spleen, liver, and kidneys, and was well detectable in the cardiovascular system for up to 21 h after injection. The time course of blood clearance was determined in vivo by gamma counting of small blood samples after IV injection (Fig. 3B) and subcutaneous injection (Fig. 3C). Blood half-life after IV injection was 13.1 h (asymmetrical confidence interval, CI: 10.81 to 15.67, one-phase exponential decay model of averaged blood activity, 6 mice). The blood signal of ^111^In-DTPA-XTEN864-anxA5 after subcutaneous injection was fitted with a two-compartment model {Y = A*[exp(− K2*t) − exp(− K1*t)]} of mean activity, 5 mice) and resulted in rate constants of 0.364 (CI: 0.2305 to 0.5850) for K1 and 0.0271 (Ci: 0.0200 to 0.0375) for K2. The biodistribution of ^111^In-DTPA-XTEN864-anxA5 four days after injection was quantified ex vivo by gamma counting (Fig. 4) and corresponded the results presented in Fig. 3A. The highest ^111^indium-specific activity per gram tissue was measured in the liver, spleen, and kidney. Accumulation in other organs was relatively low. Interestingly, there was still activity in blood and, as expected, activity was higher after subcutaneous injection.

### XTEN864-anxA5 is mainly found in the adventitia of advanced atherosclerotic lesions of ApoE knockout mice

**Fig. 5:**
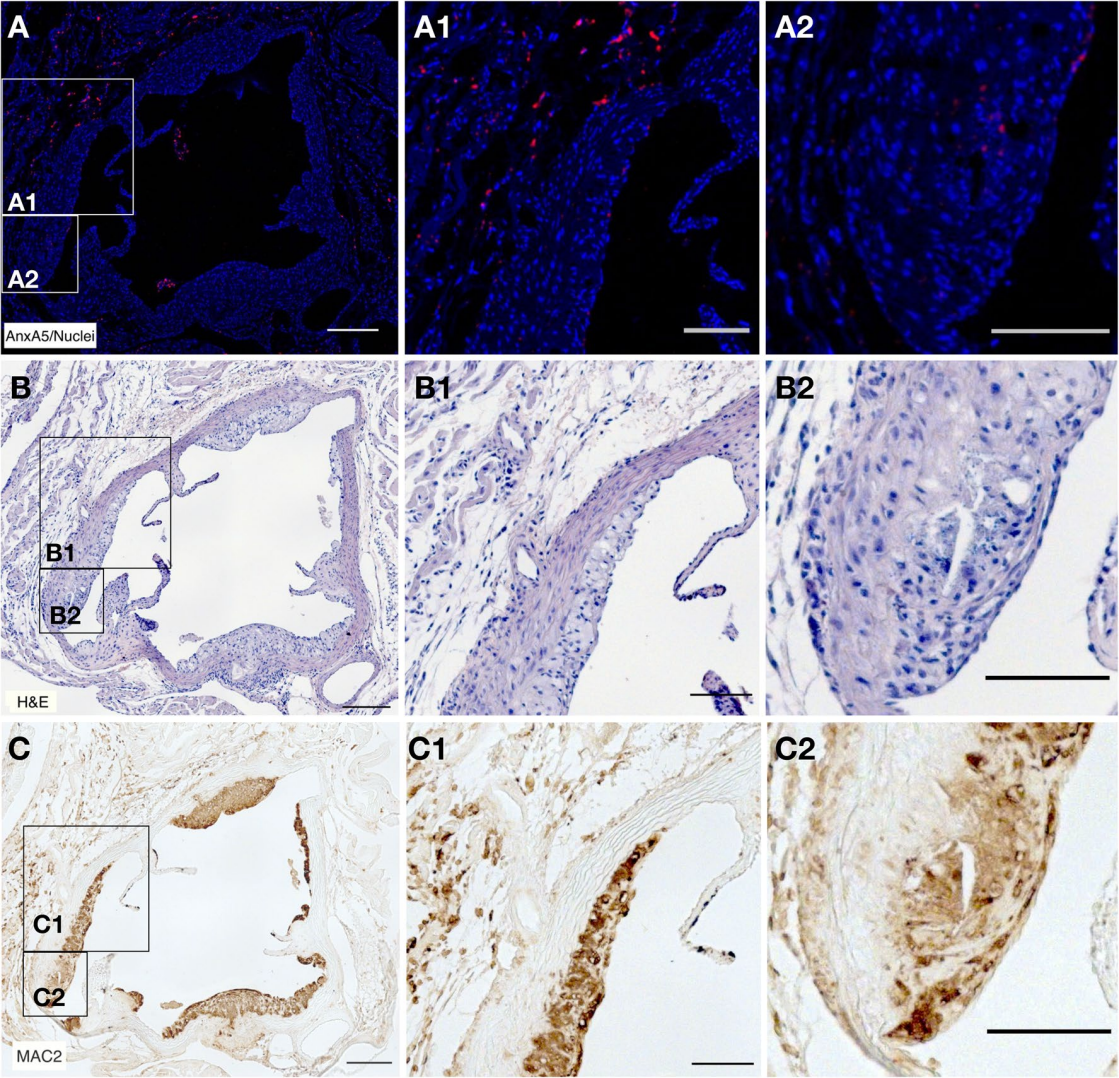
Tissue distribution of MeCAT(Ho)-XTEN864-anxA5 targeting of atherosclerotic plaques by imaging mass spectrometry and conventional immunohistology. **A** Holmium-labeled MeCAT(Ho)-XTEN864-anxA5 (red) was intravenously administered to atherosclerotic ApoE knockout mice, and tissue sections of atherosclerotic plaques in the aortic root region were imaged together with nuclei (DNA intercalator Ir-193, blue) using a Hyperion imaging mass spectrometry system. **B** Hematoxylin and eosin (H&E) stain of a parallel section of (**A**). **C** Conventional micrographs of a parallel section of (**A**, **B**) stained with anti-Mac-2 macrophage antibodies (brown). Examination of aortic root section reveals accumulation of MeCAT(Ho)-XTEN864-anxA5 (**A**) mainly in the adventitia and less pronounced accumulation in the thickened intima. Scale bars: A-C - 200 µm; A1/2-C1/2 - 100 µm (Color figure online)

To demonstrate targeted accumulation, we labeled XTEN864-anxA5 with MeCAT containing complexed holmium, a rare-earth metal not present in vivo that allows specific detection by imaging mass spectrometry. MeCAT(Ho)-XTEN864-anxA5 was intravenously administered as a single injection to ApoE knockout mice fed with high-lipid diet to demonstrate its targeting to advanced atherosclerotic plaques. Twenty-four hours after injection, aortic root sections were removed and fixed. The resulting tissue sections were counterstained with a ^193^Iridium nuclear stain and imaged using a Hyperion imaging system, which combines CyTOF technology and Imaging Mass Cytometry (IMC) (Fig. 5A). Comparison with H&E (Fig. 5B) and Mac-2 macrophage antibody (Fig. 5C) stained parallel sections demonstrated that MeCAT(Ho)-XTEN864-anxA5 mainly accumulated in the adventitia of advanced atherosclerotic plaques, where we also observed macrophages. Less MeCAT(Ho)-XTEN864-anxA5 was detectable in areas of thickened intima (Fig. 5A2) with high amounts of macrophages (Fig. 5C2). In contrast, an aortic section with normal morphology of the descending thoracic aorta, which is a region without high prevalence of plaque formation [45], revealed minimal macrophage content and almost no XTEN864-AnxA5 (see Supplementary material Fig. S1).

**Fig. 6:**
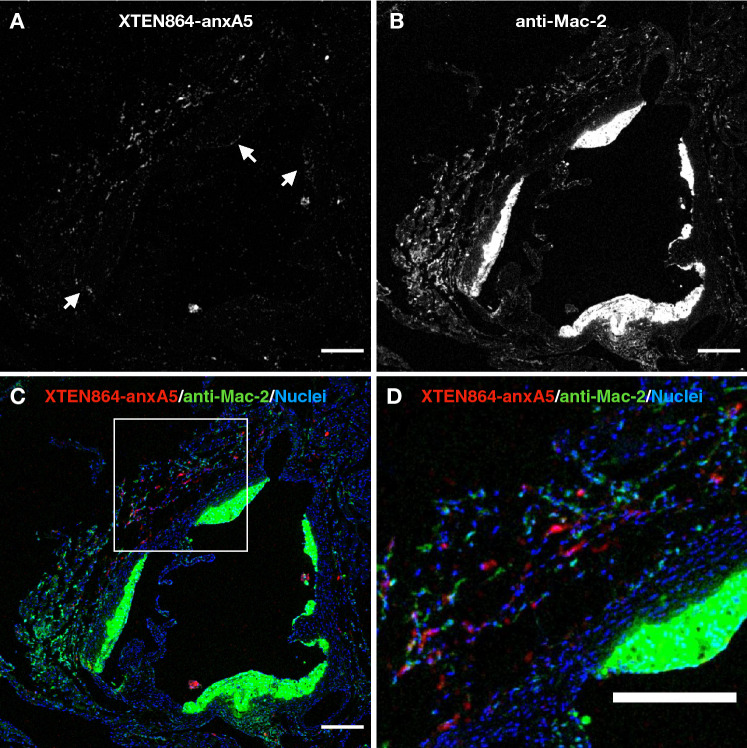
Direct correlation of IV MeCAT(Ho)-XTEN864-anxA5 in atherosclerotic plaques with metal-labeled macrophage antibodies staining by Hyperion imaging mass spectrometry. As in Fig. 5, MeCAT(Ho)-XTEN864-anxA5 (red, **A**, **B**) accumulated mainly in the adventitia and less markedly in the pathologically thickened intima. Overlays show that MeCAT(Ho)-XTEN864-anxA5 signals are not overlapping with the macrophage stain (thulium-labeled anti-Mac-2 macrophage antibodies), while MeCAT(Ho)-XTEN864-anxA5-positive areas also contain substantial amounts of macrophages. Some weakly positive stain is seen in plaque shoulders and thickened intima (**C**, arrows), overlaid with strong Mac-2 signal. Bars, 200 µm (Color figure online)

To observe MeCAT(Ho)-XTEN864-anxA5 accumulation and macrophage distribution in the same tissue section by imaging mass spectrometry, anti-Mac-2 macrophage antibodies were labeled with thulium-containing MeCAT. The MeCAT(Ho)-XTEN864-anxA-containing tissue sections were stained with MeCAT(Tm)-Mac-2 antibodies and ^193^Iridium as nuclear stain and imaged with the Hyperion system (Fig. 6). Figure 6A, B show similar distribution patterns as demonstrated by conventional immunohistochemistry (Fig. 5C). The three-channel Hyperion micrographs in Fig. 6C, D reveal that MeCAT(Ho)-XTEN864-anxA5 accumulates in plaque areas that contain macrophages without showing a direct overlap. As for Fig. 5, intimal segments with very strong macrophage signal contain only small amounts of MeCAT(Ho)-XTEN864-anxA5.

## Discussion

The purpose of this study was to develop an anxA5 fusion protein based on XTEN with very long blood circulation for long-lasting therapeutic effects. In an earlier study, we presented a fusion protein termed XTEN288-anxA5, which was developed for molecular imaging and has a blood half-life of one hour [36]. For XTEN864-anxA5, anxA5 was fused to a long version of XTEN consisting of 864 amino acids. For detection, a single cysteine-coupling site at the N-terminal end of XTEN864-anxA5 was efficiently labeled with thiol-reactive maleimide compounds: with the NIRF fluorescent dye 6S-IDCC, a Me-CAT-Holmium label for imaging mass spectrometry, and with DTPA for radionuclide labeling. However, ^111^Indium labeling of the DTPA-XTEN864-anxA5 resulted in a smaller yield compared with DTPA-XTEN288-anxA5, while ^111^In-labeling of a DOTA-XTEN864-anxA5 variant was even less successful (data not shown). XTEN864 contains 17% negatively charged glutamic acid residues, which serve as a powerful purification tag, but might cause substantial weak unspecific complexion of ^111^In during the labeling procedure. Such complexes are washed away during size exclusion purification. The selectivity of XTEN864-anxA5 for apoptotic cells is not impaired by XTEN and comparable to commercial cys-anxA5-FITC, as shown by flow cytometry.

The very short blood half-life of less than 7 min of anxA5 [46] was extended to 13 h for XTEN864-anxA5 in mice. Based on what we know from other XTEN fusion proteins, this could translate to a blood half-life of 3 days in humans [37], which in turn would ensure therapeutic blood levels for long-term treatment. In comparison, a homodimer of annexin A5 (diannexin) was reported to increase blood half-life in rats from 20 min to 6.5 h after IV injection[47]), which might translate to an estimated 12 h in humans [37]. Moreover, diannexin was shown to protect against renal, lung, pancreatic, and hepatic ischemia reperfusion injury (IRI) in mice [47–50] and might thus reduce rejection responses of organ transplants. It also reduced infarct size within hours following severe myocardial ischemia in rabbits [51] by suppression of inflammatory responses and was helpful in critical conditions such as sepsis and hemorrhagic shock [30, 31]. Besides diannexin, the blood half-life of anxA5 has been extended to about 5 h in mice by chemically coupling multiple PEG chains via the amine groups of anxA5 [52]. This approach includes the risk of substantial deactivation of the anxA5 binding affinity to apoptotic cells [53], which was not thoroughly investigated by the authors [52]. In contrast, XTEN is fused to the N-terminus of anxA5, which is opposite to the binding plane and labeling procedures were done site specifically at the cysteine of the N-terminal end of XTEN.

In addition, phosphatidylserine was identified as an important immune checkpoint that influences immune responses directed against tumor cells and tumor-associated macrophages (TAM) that modulate the tumor microenvironment (TME) and upregulate the expression of TAM receptor tyrosine kinases (Tyro3, Axl, and MerTK) that interact with PS. This promotes TGF-β secretion, inhibits TNF-α production by innate immune phagocytes inside the TME, and suppresses proper function of CD8+ T cells [4, 6–8]. Moreover, it was shown that the ROS (reactive oxygen species) activity and hypoxic conditions of the TME cause apoptosis of regulatory T cells (Tregs), and this is a strong immunosuppressive effect within the TME, partly mediated via the subsequent uptake and processing by phagocytic macrophages[54].

Another remarkable study recently published by Kang et al*.* postulates that anxA5 treatment may enhance the T-cell CD8+ response by binding PS and thus suppressing the immunosuppressive effects of Tregs or by inhibiting interactions between apoptotic Tregs and phagocytes in the TME [32]. The results of this study thus suggest that anxA5 has the potential to inhibit chemotherapy-induced immune suppression in the TME in an animal model. Moreover, administration of anxA5 combined with chemotherapy or tumor vaccine (specific tumor-derived peptides) was shown to strongly improve the outcome of treatment, and only the combination of all three resulted in tumor regression and survival. However, the authors also pointed out the short physical half-life of anxA5, which may complicate routine clinical treatment despite the stronger binding affinity of anxA5 to PS compared with anti-PS antibodies.

As mentioned above, XTEN864-anxA5 potentially has very good properties to accumulate in tumor and its TME through the high-affinity binding to PS, the enhanced permeability and retention (EPR) effect when present [55], and the very long blood circulation time. These effects can be advantageous not only for tumor treatment but also for the therapy of infectious diseases. Various biological agents such as viruses, bacteria, and parasites use apoptotic mimicry by exhibiting PS on their surface to infect host cells and escape clearance. Furthermore, they can upregulate PS in infected cells or surrounding cells. The deceptive presentation as apoptotic debris promotes phagocytic uptake and therefore subsequent infection as well as suppression of rapid elimination by T-cells [4, 20, 56]. Effective and high-affinity binding of XTEN864-anxA5 to PS in conjunction with a sufficiently long circulation time and a two-dimensional crystallization shield effect by anxA5[57] as well as a hydrodynamic covering effect by the XTEN864 polypeptide are desirable properties for treating infectious processes.

Beyond its potential benefit in the treatment of infection and cancer, long-circulating XTEN864-anxA5 may also have a role in treating cardiovascular diseases. Our results in advanced plaques of ApoE^−/−^ mice show accumulation of XTEN864-anxA5 in macrophage-positive compartments of the adventitia in contrast to a normal appearing aortic section, which supports its potential to attenuate inflammation (Figs. 5, 6, S1). Stoer et al. [27] and Burgmayer et al. [26] demonstrated that anxA5 treatment reduced the amount of macrophages in different stages of plaque development, but attenuated plaque size and the amount of apoptotic cells only in early stages. The adventitia—the outer layer of the vessel wall—is a collagen-rich extracellular matrix with bunches of fibroblasts, perivascular nerves, and microvessels. More recent studies suggest that the adventitia plays a very complex and dynamic role in the growth, repair, and diseases of the arterial wall [58]. Healthy adventitia contains various cell types involved in the control and modulation of the immune response such resident macrophages, mast cells, T and B lymphocytes, and dendritic cells, as well as stem/progenitor cells [59]. A microvascular network in the adventitia—the vasa vasorum (VV)—supplies the arterial wall with all necessary biomolecules and oxygen from blood while at the same time providing a niche for the bidirectional migration through the vessel wall of inflammatory macrophages, leukocytes, and myofibroblasts during pathogenesis [60], e.g., in response to hyperlipidemia. Recent studies of atherosclerosis development in different animal models reveal high heterogeneity of immune cells in the adventitia and strongly support the “outside-in” theory of vascular inflammation, which begins in the adventitia and advances towards the media and intima, causing plaque formation with increasing activation of resident macrophages, attraction of immune cells, and expansion of VV [58, 60, 61]. According to Butcher et al. [62], resident-like macrophages, which are most abundant in the adventitia, are unable to phagocytose oxidized low-density lipoprotein (LDL) efficiently, but are highly effective efferocytes that secrete a variety of matrix metalloproteases and could thus destabilize advanced plaques. Deng et al. have demonstrated that inflammatory and resident-like macrophages make up 70% of the macrophage populations after 20 weeks of high-fat diet in LDL(-) mice, which promotes progression of atherosclerosis [63].

A study by de Jong et al. demonstrated rapid accumulation of administered anxA5 in the infarcted area, resulting in a reduction of infarct size and a decrease in the number of cardiac macrophages. [29] Further, three weeks after myocardial ischemia and reperfusion injury, the authors observed reduced dilation of the left ventricle and improved cardiac function, despite rapid clearance of anxA5 from blood. Ewing et al. also reported dose-dependent effects of anxA5 treatment after vascular injury by preventing leukocyte recruitment and atherosclerosis development [28]. We speculate that, with the longer blood circulation and the shielding effects of XTEN, XTEN864-anxA5 might have an improved therapeutic potential in the treatment of acute vascular ischemia and reperfusion injuries in comparison to wild-type anxA5 and diannexin.

Finally, in patients with anti-phospholipid syndrome (APS), circulating aPL (anti-phospholipid) antibodies disrupt the anticoagulant crystallization shied of anxA5 and consequently highly promote thrombosis in the placenta and the cardiovascular system [64–67]. PS autoantibodies might be also responsible for the incased thrombotic risk in COVID-19 [68]. Bound XTEN864-anxA5 could potentially counter these antibodies by shielding activated platelets with its large XTEN-bound hydrate shell and thus restore hemostatic balance.

## Conclusion

XTEN864-anxA5 extends the blood half-life of AnxA5 from 7 min to 13 h in mice, while retaining its binding affinity to PS-exposing membranes. With an estimated blood half-life of 3 days in humans, XTEN864-anxA5 has promising therapeutic properties for treating a wide range of acute disease states that involve exposure of PS. Possible therapeutic applications include prevention of thrombosis and thromboembolic states, counteracting immunosuppression in the tumor microenvironment, and disruption of viral replication by blocking apoptotic mimicry of highly dangerous viruses and other germs. Besides prolongation of blood circulation, the fusion of the large XTEN864 polypeptide with its extensive hydrate shell should add unspecific shielding properties with regulative potential of its own to AnxA5.

## Supplementary Information

Below is the link to the electronic supplementary material.

Supplementary file1 (PDF 2237 kb)

## Data Availability

All data, materials and applied software support our published claims and comply with field standards. The amino acid sequence of XTEN864-anxA5 is provided as supplement. Further datasets generated during and/or analyzed during the current study are available from the corresponding author on request.
