# Supplementary material for: Long-circulating XTEN864-annexin A5 fusion protein for phosphatidylserine-related therapeutic applications
Source: Apoptosis. 2021 Aug 17;26(9-10):534–47. doi: 10.1007/s10495-021-01686-w (PMC8370750; doi:10.1007/s10495-021-01686-w)
Supplement: Supplementary file 1 — Supplementary file1 (PDF 2237 kb) [file 10495_2021_1686_MOESM1_ESM.pdf]

Supplementary information

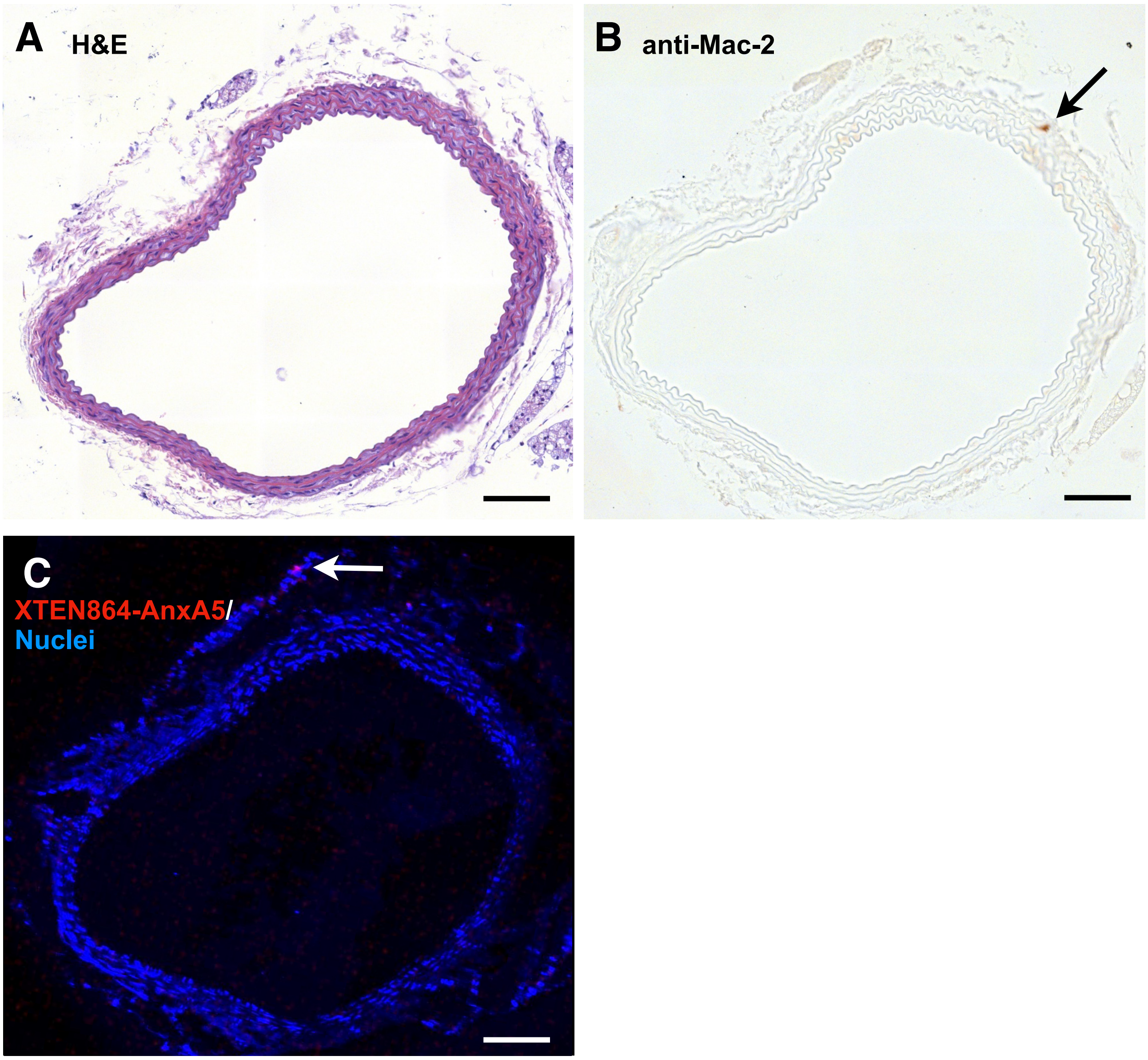

Figure S1: Distribution of XTEN864-AnxA5 in aortic tissue section without signs for atherosclerotic lesions. (**A**) Tissue section with H&E staining of the descending thoracic aorta of ApoE knockout mouse without signs of atherosclerotic plaques. (**B**) Consistent with the normal morphology, Mac-2 stain for macrophages was only scarce (arrow), ruling out significant inflammation. Corresponding to the histology, a Hyperion™ image (**C**) of a parallel section revealed only scarce accumulation of XTEN864-AnxA5 (arrow). Scale bar 100 µm.

Protein sequence of the XTEN864-AnxA5 fusion protein

CGSPAGSPTSTEEGTSESATPESGPGTSTEPSEGSAPGSPAGSPTSTEEGTSTEPSEGSAPGTSTEP
SEGSAPGTSESATPESGPGSEPATSGSETPGSEPATSGSETPGSPAGSPTSTEEGTSESATPESGPGT
STEPSEGSAPGTSTEPSEGSAPGSPAGSPTSTEEGTSTEPSEGSAPGTSTEPSEGSAPGTSESATPES
GPGTSTEPSEGSAPGTSESATPESGPGSEPATSGSETPGTSTEPSEGSAPGTSTEPSEGSAPGTSES
ATPESGPGTSESATPESGPGSPAGSPTSTEEGTSESATPESGPGSEPATSGSETPGTSESATPESGPG
TSTEPSEGSAPGTSTEPSEGSAPGTSTEPSEGSAPGTSTEPSEGSAPGTSTEPSEGSAPGTSTEPSE
GSAPGSPAGSPTSTEEGTSTEPSEGSAPGTSESATPESGPGSEPATSGSETPGTSESATPESGPGSEP
ATSGSETPGTSESATPESGPGTSTEPSEGSAPGTSESATPESGPGSPAGSPTSTEEGSPAGSPTSTEE
GSPAGSPTSTEEGTSESATPESGPGTSTEPSEGSAPGTSESATPESGPGSEPATSGSETPGTSESATP
ESGPGSEPATSGSETPGTSESATPESGPGTSTEPSEGSAPGSPAGSPTSTEEGTSESATPESGPGSEP
ATSGSETPGTSESATPESGPGSPAGSPTSTEEGSPAGSPTSTEEGTSTEPSEGSAPGTSESATPESGP
GTSESATPESGPGTSESATPESGPGSEPATSGSETPGSEPATSGSETPGSPAGSPTSTEEGTSTEPSE
GSAPGTSTEPSEGSAPGSEPATSGSETPGTSESATPESGPGTSTEPSEGSAPAQVLRGTVTDFPGFER
ADAETLRKAMKGLGTDEESILTLLTSRSNAQRQEISAAFKTLFGRDLLDDLKSELTGKFEKLIVALMKPS
RLYDAYELKHALKGAGTNEKVLTEIIASRTPEELRAIKQVYEEEYGSSLEDDVVGDTSGYYQRMLVVLL
QANRDPDAGIDEAQVEQDAQALFQAGELKWGTDEEKFITIFGTRSVSHLRKVFDKYMTISGFQIEETID
RETSGNLEQLLLAVVKSIRSIPAYLAETLYYAMKGAGTDDHTLIRVMVSRSEIDLFNIRKEFRKNFATSLY
SMIKGDTSGDYKKALLLLSGEDDGG

Cysteine – yellow
XTEN – blue
Annexin A5 – green
